# Beyond the clot: how the biomolecular landscape of platelet-rich fibrin directs fibroblast functions

**DOI:** 10.3389/froh.2025.1731949

**Published:** 2026-01-20

**Authors:** Jiarui Bi, Hannu Larjava, Lari Häkkinen

**Affiliations:** Faculty of Dentistry, Department of Oral Biological Sciences, University of British Columbia, Vancouver, BC, Canada

**Keywords:** fibroblast functions, inflammation, platelet-rich fibrin, PRF, regenerative therapies, soft tissue repair, wound healing

## Abstract

Platelet-Rich Fibrin (PRF) is a second-generation autologous blood concentrate widely applied in regenerative medicine and dentistry for its wound-healing potential. Its clinical applications span dermatology, plastic surgery, periodontology, implantology, and oral maxillofacial surgery, with growing evidence supporting its effectiveness in tissue regeneration. Fibroblasts, as central regulators of extracellular matrix synthesis and remodeling, angiogenesis, and inflammation, are important targets of PRF's regenerative effects. This review summarizes the recent evidence of role of PRF in regulation of fibroblast functions important for wound healing and inflammation. It highlights PRF as a biologically active scaffold that accelerates soft tissue repair, primarily through modulation of fibroblasts, positioning it as a promising adjunct in regenerative therapies.

## Introduction

Platelet-Rich Fibrin (PRF) is an autologous biomaterial produced from the patient's venous or arterial blood. It is now recognized for its potential in regenerative medicine and dentistry. PRF is a second-generation blood concentrate derived from the original platelet-rich plasma (PRP) (1). Roles of various blood concentrates in clinical applications have been extensively investigated in the literature. For instance, they have been used to promote healing of compromised wounds, including chronic skin ulcers, necrosed/infected wounds and chronic osteomyelitis. They are also used in plastic surgery in different ways, such as topical applications or injections in various skin rejuvenation treatments, lip augmentation, treatment of nasolabial fold defects, acne scars, and wrinkles (2–4). However, controlled clinical trials in their use in medicine are still limited and need further investigation. In dentistry, particularly PRF has gained interest in various procedures in periodontology, oral maxillofacial surgery, implantology, and others. A recent meta-analysis showed that PRF achieves better pocket reduction and clinical attachment gains compared to open flap debridement/bone graft alone in treating periodontal defects (5). A meta-analysis of PRF in the treatment of medication-related osteonecrosis of the jaw also favored the use of PRF alone or combined with other therapies, with high resolution of the lesion after PRF therapy compared to the other approaches (6).

PRF, in its various forms, has become a rapidly growing area of research in recent years. A search of PubMed using the keywords “[PRF(Title)] OR [Platelet-rich fibrin(Title)]” retrieves over 2,100 publications from the past decade, including more than 200 review articles, reflecting the increasing scientific interest and clinical relevance of PRF. PRF is widely used as a membrane to cover the healing sites of surgeries, where it comes into close contact with connective tissue. Thus, the wound healing promoting effects of PRF are mediated by interactions of cells and bioactive molecules present in PRF with the surrounding tissue cells. Fibroblasts, as the primary stromal cells of the connective tissues, play crucial roles in tissue repair by migrating to the wound site, regulating inflammation and angiogenesis, producing extracellular matrix (ECM), and contracting and remodeling the wound. This review examines the recent evidence on the biological effects of PRF on fibroblasts.

## What is PRF?

During wound healing, normal blood clot formation is not only important to stop bleeding, but also the cells and molecules present in the clot are crucial to kickstart the wound healing process. Therefore, it is conceivable that applying a biomaterial that contains a concentrate of key components of the clot, such as PRF, to the wound site would be beneficial for wound healing. The preparation of PRF involves collecting the patient's blood, which is then subjected to centrifugation without the use of anticoagulants. This activates the coagulation cascade of the collected blood leading to a relatively slow formation of a fibrin clot that traps various molecules from circulating blood and most of the platelets, while leukocytes and red blood cells are removed from the concentrate. In a widely used modification of PRF, called leukocyte- and platelet-rich fibrin (L-PRF), leukocytes are also retained in the final product (1). Generation of PRF by centrifugation leaves behind a supernatant that contains plasma components that are not brought down by centrifugation and incorporated into the PRF clot. The composition of this supernatant (called platelet poor plasma, PPP) is distinct from the PRF itself, and can also be used separately for certain regenerative purposes in a liquid form (1). During preparation of PRF, platelets and leukocytes become activated and release an array of bioactive molecules into the PRF matrix (7–9). In comparison to the original PRP, which was generated by adding clotting accelerators (usually calcium chloride) to quickly form the clot, the more slowly polymerizing PRF clot is structurally stronger and can be molded to be used as a bioactive membrane that degrades and releases bioactive molecules more slowly compared to PRP (9). However, factors like centrifugation force and time, tube angulation, and delayed centrifugation can affect the quality and size of the PRF (1, 10). For instance, a lower speed and horizontal angulation results in more evenly distributed cell components and greater separation of blood layers (1). In addition, a 6-minute delay of centrifugation leads to about 30% reduction in the mass weight of PRF compared to non-delayed protocol (10). Furthermore, use of glass tubes produces higher-weight PRF membranes compared to silica-coated plastic tubes (11). Finally, differences in PRF preparations between arterial and venous blood have been reported (12). Thus, depending on preparation methods, PRF composition and properties may vary, complicating interpretations of studies exploring its properties *in vitro* and *in vivo*.

Detailed studies have shown that in general the PRF clot can contain over 95% of the platelets and 50% of the leukocytes from the collected blood. It also has a matrix with high fibrin content, as expected, and a variety of soluble components entrapped in the matrix (8, 13). These bioactive molecules could include hormones, chemokines, cytokines, growth factors and proteolytic enzymes normally present in blood circulation as well as molecules released from activated platelets and leukocytes (13, 14). It is likely that one of the key advantages of PRF membranes over other autologous blood preparations is that they can provide sustained release of these bioactive molecules over several days that span the early stages of wound healing (7, 14). This is supported by *in vitro* studies showing that depending on preparation method, PRF membranes can maintain their integrity for more than seven days (9). The release rate of the bioactive molecules from PRF may also vary depending how PRF is prepared. For instance, a delay in blood centrifugation may result in a lower releasable protein concentration (10). A recent protocol of PRF preparation, called albumin-platelet-rich fibrin (Alb-PRF), can also greatly extend the degradation time of PRF, and accordingly release rate of certain growth factors, including platelet-derived growth factor (PDGF), transforming growth-factor beta-1 (TGF-β1), vascular-endothelial growth factor (VEGF), insulin-like growth factor (IGF) and epidermal growth factor (EGF), up to 10 days. This protocol involves separating platelet-poor plasma from the collected blood followed by denaturation of albumin by heating, and then mixing it back with the cell-rich proportion (15, 16). Further development of PRF preparation protocols may provide further optimized bioactive membranes for various clinical uses. More in depth understanding of the composition and biological effects of PRF on key cells involved in wound healing will also help to achieve this goal.

## Identity of fibroblasts

Various tissues in human body show different regeneration potential. For instance, oral mucosal wounds associate with a faster closure speed and relatively scarless healing, which is distinct from skin wounds (17). At histological level, intraoral wounds also exhibit faster re-epithelialization, resolution of inflammation and angiogenesis. This may in part depend on the properties of oral mucosal fibroblasts as they appear to be preprogrammed to support rapid resolution of inflammation and secrete factors that promote re-epithelialization, angiogenesis and ECM remodeling. In contrast, skin fibroblasts tend to produce abundant ECM, exhibit active TGF-β signaling, and have more contractile properties, often leading to scar formation and fibrosis (18). Thus, fibroblasts in different tissues have different properties that may be important also for the wound healing outcome. However, the emerging narrative maybe even more complicated. Recent advances from studies using single cell RNA-sequencing (scRNA-seq) in human and animal studies and lineage tracing in various animal models have shown that the role of fibroblasts in tissue repair may depend on different fibroblast subpopulations found in the tissues. These subpopulations are dynamic and show different functions and identities, which sometimes overlap with those with mesenchymal stem cells (MSC). *In vivo* these subpopulations can be distinguished based on e.g., developmental origin, anatomical location, gene expression signature and functional state. The different subpopulations can be pro- or anti-inflammatory, can interact distinctly with immune cell subsets, may promote angiogenesis, can be actively involved in ECM synthesis and remodeling, but also can contribute to pathologies such as fibrosis (19–23). For instance, based on studies using scRNA-seq technique, lineage tracing and other approaches, oral mucosa has been shown to contain fibroblast subpopulations that can be distinguished by expression of distinct genes, including paired-related homeobox-1(*Prx1*) and angiotoxin receptor-like (*Axl*), that associate with their immunomodulatory and pro-regenerative phenotypes (24–26). Conversely, oral mucosa contains few fibroblasts that can be defined by expression of Engrailed-1 (*En1*) and dipeptidyl peptidase 4 (*Dpp4*, CD26), which contribute to scar formation in skin (27, 28). Although our understanding is still incomplete, it appears that activation of these subpopulations is distinctly regulated by the signals that are present in the wound site (29–32). Therefore, different fibroblast subsets maybe also regulated in a tissue-specific manner by bioactive components present in PRF. Detailed understanding of fibroblast subpopulations within each tissue and composition of PRF would allow to produce tailored PRF preparations that best match the cellular composition of the target tissue.

## Role of fibroblasts in wound healing

Fibroblasts in general play several key roles throughout the wound healing cascade by producing various molecules to regulate functions of other cells, by modifying the local environment and restoring the tissue integrity (33). Specifically, proper activation and dynamic spatiotemporal regulation of fibroblast subpopulations after injury is required for these cells to appropriately participate at the early inflammation, proliferation and granulation tissue formation stages of wound healing (at day 1–14 after injury), and to accomplish subsequent tissue remodeling important for tissue regeneration (34). Based on degradation and growth factor/cytokine release kinetics of PRF (1) it appears that PRF applied to the wound particularly facilitates these early wound healing stages. It is conceivable that the composition, concentration and duration of activity of factors released from PRF over these first two weeks maybe important to guide the wound healing process into a pathway of regeneration.

During normal wound healing, fibroblast activation occurs within the first 24–48 h after injury and is partially triggered by soluble mediators such as chemokines, cytokines, and growth factors, from the blood clot and inflammatory cells. During the first days after injury, activated fibroblasts proliferate and migrate into wound provisional fibrin-rich matrix (34, 35). These fibroblasts also reciprocally regulate inflammation by producing proinflammatory cytokines and chemokines, such as tumor necrosis factor-alpha (TNF-α), interferon gamma (IFN-γ), interleukin-6 (IL-6), interleukin-12 (IL-12), C-X-C Motif Chemokine Ligand 1 (CXCL1), C-X3-C motif chemokine ligand 1 (CX3CL1), and C-C motif chemokine ligand 2 (CCL2, monocyte chemoattractant protein-1), to further attract immune cells to the wound site (36, 37). These immune cells, including macrophages, produce TGF-βs, PDGF, interleukin −1 (IL-1), fibroblast growth factor (FGF), and TNF-α (38–40) that further facilitate the repair process. They also induce fibroblasts to produce VEGF, FGF, angiopoietin 1 (Ang-1), thrombospondins (THBSs), CXCL12 (stromal cell-derived factor-1a), CXCL8 (IL-8), and CCL2 to promote angiogenesis and to facilitate formation of granulation tissue (41, 42). The factors secreted by macrophages, such as TGF-βs, stimulate fibroblasts to produce various ECM molecules such as collagens, fibronectin, hyaluronic acid, and proteoglycans, to support cell migration into the fibrin clot and serve as initial ECM that gradually replaces the fibrin clot with granulation tissue (34, 35). Fibroblasts also support re-epithelialization by secreting keratinocyte growth factor (KGF/FGF7) and hepatocyte growth factor/scatter factor (HGF/SF). After about 7–10 days (depending on wound size), fibroblasts in the granulation tissue differentiate into myofibroblasts that, while actively secreting ECM, contract the wound to reorganize the ECM (43, 44). These events at the early stages of the healing are critical for subsequent remodeling stage that normalizes the tissue structure and function, and is largely accomplished by fibroblasts subpopulations that express a set of genes that are believed to be important for tissue remodeling (34, 35). It is not completely clear whether wound healing is accomplished by a dynamic emergence and disappearance of different fibroblast subpopulations or plasticity of the same populations over the course of healing, or a combination of both. In any case, delay in early wound healing stages can lead to chronic wounds or fibrosis (pathological scar formation), which may be linked to persistence or activation of tissue-specific fibroblast subpopulations that do not support appropriate tissue regeneration (33). To summarize, by crosstalk with various cells and by producing and organizing wound ECM, fibroblasts orchestrate the function of other cells needed for effective healing. Therefore, any therapeutic modality that is expected to promote tissue regeneration should be composed of bioactive molecules that promote these functions of fibroblasts in a spatiotemporally appropriate manner.

## Composition of PRF

Composition of PRF preparations derives from blood plasma, platelets and leukocytes (particularly granulocytes, T-cells, B-cells, NK/T cells, and monocytes), and molecules released by these cells (1, 12). Human plasma itself contains more than five thousand circulation proteins (45) and a variety of extracellular vesicles (EVs) released from various cells and tissues in the body (46). EVs are membrane-coated, nano meter-range vesicles that contain various growth factors, nucleic acids and lipids that can have pro-regenerative signaling functions on target cells (46, 47). The composition of plasma between individuals may vary pending on e.g., sex, age, health status and use of medications (45, 48). Therefore, it can be expected that PRF preparations are a composite of various bioactive molecules that may also vary between different individuals. The force used to generate PRF preparations by centrifugation is typically 400–700 G (49). This relatively low force allows to maintain live cells intact, but is not sufficient to concentrate small, low molecular weight molecules, such as certain hormones, cytokines, cytokines, growth factors or EVs (which may require a ten-fold or higher G-force) from plasma into the final PRF matrix. It is expected that only those small molecules and EVs that have the ability to bind with the larger molecules, including fibrinogen/fibrin or fibronectin, may be captured in the PRF matrix during centrifugation. Therefore, the majority of the EVs and small molecules found in PRF are likely secreted by the cells (platelets and leukocytes) within PRF (50). Contact with the hydrophobic surface of the blood collection tube, centrifugation and blood coagulation activates these cells leading to a fast release of their cargo (e.g., EVs, soluble chemokines, cytokines, growth factors and proteases) (48, 51). Some of the cells also survive in PRF for several days and gradually synthesize and release these factors over time. Based on culture experiments of PRF and analysis by histology, fluorescence activated cell sorting (FACS), scRNA-seq and PCR, it appears that T-cells, B-cells and macrophages, likely differentiated from monocytes, are still present after 7 days, while some of the other immune cells seem to decrease in abundance, particularly granulocytes that have a reported life span of only 6 h. The shift in cell populations associates with a change in release of certain bioactive molecules over time, and, interestingly, a sustained release of macrophage-associated growth factors TGF-β1, PDGF and VEGF (12). However, when released from cells or matrix storage, growth factors and cytokines can be very short-lived. Half-life of active soluble TGF-β1 is only 2–3 min while its matrix-bound form can last at least for 90 min (52). Therefore, for sustained release, PRF needs to maintain cells metabolically active to produce and release these factors for an extended period and contain proteins that are able to bind, store and protect the released molecules. Although proteomics analysis of PRF has improved greatly during the recent years, we have not likely completely captured the complexity of this biomaterial yet.

As mentioned above, during PRF preparation, blood coagulation induces polymerization of fibrinogen into a 3-dimensional fibrin-rich network that contains concentrated cells and bioactive molecules from the blood (1, 48). In order to understand the composition of PRF, studies have analyzed it by various immunoassays (immunoblotting and enzyme-linked immunosorbent assays, ELISA) (13, 14, 51, 53–55). These relatively sensitive approaches usually look for few preselected components, particularly matrix molecules that are suspected to be present, and cytokines and growth factors, which are known to be released by activated platelets and leukocytes. While the blood donor characteristics, PRF preparation and analysis methods often vary, in general these studies have confirmed that PRF contains a mixture of matrix molecules, including fibronectin, vitronectin and thrombospondin-1(TSP-1), in addition to fibrin, that not only provide structural integrity for PRF but are known to support fibroblast adhesion and migration to the site. It also contains and gradually releases soluble molecules, such as PDGF, TGF-β1, VEGF, bone morphogenic proteins (BMPs), FGF2, EGF, and IGF1 (7, 56, 57).

With the advancement of proteomic technologies, recent studies have employed non-biased high-resolution mass spectrometry (MS/MS) to comprehensively profile the PRF proteome. A targeted literature search identified four publications that provided primary MS-based protein profiling data for review (Table 1). Usually, these studies examine either soluble molecules released from the PRF into a liquid medium (releasate) over a period of time or homogenize the entire PRF membrane to analyze the matrix, entrapped molecules and cells at the same time. Yaprak et al. (58) used time-of-flight (Tof/Tof) MS/MS to study releasate collected immediately after preparation of the PRF (produced by centrifugation with 400 G for 10 min) and detected 35 common proteins from all 8 samples from healthy donors, including 16 of them that were linked to the wound healing process (58). Among all 35 proteins, 31 proteins were also shared with proteomic datasets from other studies (59–61). These included protease inhibitors (α1-antitrypsin, α1-antichymotrypsin, haptoglobin and α2-HS-glycoprotein), immunoglobulins and complement (complement C3), apolipoproteins, and antioxidant proteins (albumin, transferrin, ceruloplasmin and hemopexin). Two publications have used liquid chromatography-tandem mass spectrometry (LC-MS/MS) to study proteins from PRF. Hermida-Nogueira et al. (59) analyzed releasate from PRF after incubation for 3, 7 and 21 days in cell culture medium and detected more than 700 proteins (59). In contrast, Di Summa et al. (61) prepared a PRF lysate by a freeze-sonication-centrifugation protocol to test the proteomic components of the entire PRF clot, and detected over 600 proteins (61). This study shared 68 common proteins with the Hermida-Nogueira et al. (59) study. Lastly, Anitua et al. (60) studied the proteome of plasma rich in growth factors (PRGF, a platelet-enriched and leukocyte-free plasma clot) by using both Tof/Tof MS/MS and LC-MS/MS, and found 49 and 167 proteins with each method, respectively (60).

**Table 1:** Common proteins identified from proteomic data across multiple MS/MS studies.

Protein names	Reported in articles	Study count	Detection method	Gene ONTOLOGY (GO) related to wound healing
6-phosphogluconolactonase (6PGL)	Anitua et al. 2015 (LS-MS/MS), Hermida-Nogueira et al. 2020, Di Summa et al. 2020	3	LC–MS/MS	
Actin, cytoplasmic 1 (Beta-actin)	Anitua et al. 2015 (LS-MS/MS & TOF/TOF MS/MS), Yaprak et al. 2018, Hermida-Nogueira et al. 2020, Di Summa et al. 2020	4	LC–MS/MS,	Chromatin remodeling [GO:0006338]; positive regulation of cell population proliferation [GO:0008284]
			MALDI TOF/TOF MS/MS	
Adenylyl cyclase-associated protein 1 (CAP 1)	Anitua et al. 2015 (LS-MS/MS), Hermida-Nogueira et al. 2020, Di Summa et al. 2020	3	LC–MS/MS	Ameboidal-type cell migration [GO:0001667]
Afamin (Alpha-albumin)	Anitua et al. 2015 (LS-MS/MS & TOF/TOF MS/MS), Yaprak et al. 2018, Hermida-Nogueira et al. 2020, Di Summa et al. 2020	4	LC–MS/MS,	
			MALDI TOF/TOF MS/MS	
Albumin	Anitua et al. 2015 (LS-MS/MS & TOF/TOF MS/MS), Yaprak et al. 2018, Hermida-Nogueira et al. 2020, Di Summa et al. 2020	4	LC–MS/MS,	
			MALDI TOF/TOF MS/MS	
Alpha-1-acid glycoprotein 1 (AGP 1; Orosomucoid-1)	Anitua et al. 2015 (LS-MS/MS), Hermida-Nogueira et al. 2020, Di Summa et al. 2020	3	LC–MS/MS	Acute-phase response [GO:0006953]; regulation of immune system process [GO:0002682]
Alpha-1-acid glycoprotein 2 (AGP 2; Orosomucoid-2)	Anitua et al. 2015 (LS-MS/MS), Yaprak et al. 2018, Di Summa et al. 2020	3	LC–MS/MS,	Acute-phase response [GO:0006953]; regulation of immune system process [GO:0002682]
			MALDI TOF/TOF MS/MS	
Alpha-1-antichymotrypsin (ACT; Serpin A3)	Anitua et al. 2015 (LS-MS/MS & TOF/TOF MS/MS), Yaprak et al. 2018, Hermida-Nogueira et al. 2020, Di Summa et al. 2020	4	LC–MS/MS,	Acute-phase response [GO:0006953]
			MALDI TOF/TOF MS/MS	
Alpha-1-antitrypsin (Alpha-1 protease inhibitor; Serpin A1)	Anitua et al. 2015 (LS-MS/MS & TOF/TOF MS/MS), Yaprak et al. 2018, Hermida-Nogueira et al. 2020, Di Summa et al. 2020	4	LC–MS/MS,	Acute-phase response [GO:0006953]; blood coagulation [GO:0007596]
			MALDI TOF/TOF MS/MS	
Alpha-1B-glycoprotein (Alpha-1-B glycoprotein)	Anitua et al. 2015 (LS-MS/MS & TOF/TOF MS/MS), Yaprak et al. 2018, Hermida-Nogueira et al. 2020, Di Summa et al. 2020	4	LC–MS/MS,	Immune response-regulating signaling pathway [GO:0002764]
			MALDI TOF/TOF MS/MS	
Alpha-2-antiplasmin (Alpha-2-AP; Serpin F2)	Anitua et al. 2015 (LS-MS/MS & TOF/TOF MS/MS), Yaprak et al. 2018, Hermida-Nogueira et al. 2020, Di Summa et al. 2020	4	LC–MS/MS,	Acute-phase response [GO:0006953]; positive regulation of coagulation [GO:0050820]; positive regulation of smooth muscle cell proliferation [GO:0048661]
			MALDI TOF/TOF MS/MS	
Alpha-2-HS-glycoprotein (Alpha-2-Z-globulin; Fetuin-A)	Anitua et al. 2015 (LS-MS/MS & TOF/TOF MS/MS), Yaprak et al. 2018, Hermida-Nogueira et al. 2020	3	LC–MS/MS,	Acute-phase response [GO:0006953]
			MALDI TOF/TOF MS/MS	
Alpha-2-macroglobulin (Alpha-2-M; C3 and PZP-like alpha-2-macroglobulin domain-containing protein 5)	Anitua et al. 2015 (LS-MS/MS), Hermida-Nogueira et al. 2020, Di Summa et al. 2020	3	LC–MS/MS	Negative regulation of complement activation, lectin pathway [GO:0001869]
Angiotensinogen (Serpin A8)	Anitua et al. 2015 (LS-MS/MS & TOF/TOF MS/MS), Yaprak et al. 2018, Hermida-Nogueira et al. 2020, Di Summa et al. 2020	4	LC–MS/MS,	Blood vessel remodeling [GO:0001974]; low-density lipoprotein particle remodeling [GO:0034374]; positive regulation of endothelial cell migration [GO:0010595]; positive regulation of extracellular matrix assembly [GO:1901203]; positive regulation of fibroblast proliferation [GO:0048146]; regulation of cell population proliferation [GO:0042127]; regulation of extracellular matrix assembly [GO:1901201]
			MALDI TOF/TOF MS/MS	
Annexin A1 (Annexin I; Calpactin II; Chromobindin-9; Lipocortin I; Phospholipase A2 inhibitory protein; p35)	Anitua et al. 2015 (LS-MS/MS), Hermida-Nogueira et al. 2020, Di Summa et al. 2020	3	LC–MS/MS	Adaptive immune response [GO:0002250]; innate immune response [GO:0045087]; myoblast migration involved in skeletal muscle regeneration [GO:0014839]; positive regulation of cell migration involved in sprouting angiogenesis [GO:0090050]; positive regulation of T cell proliferation [GO:0042102]; positive regulation of wound healing [GO:0090303]
Antithrombin-III (ATIII; Serpin C1)	Anitua et al. 2015 (LS-MS/MS & TOF/TOF MS/MS), Yaprak et al. 2018, Hermida-Nogueira et al. 2020, Di Summa et al. 2020	4	LC–MS/MS,	Blood coagulation [GO:0007596]; blood coagulation [GO:0007596]; regulation of blood coagulation [GO:0030193]
			MALDI TOF/TOF MS/MS	
Apolipoprotein A-I (Apo-AI)	Anitua et al. 2015 (LS-MS/MS & TOF/TOF MS/MS), Yaprak et al. 2018, Hermida-Nogueira et al. 2020, Di Summa et al. 2020	4	LC–MS/MS,	Blood vessel endothelial cell migration [GO:0043534]; endothelial cell proliferation [GO:0001935]; high-density lipoprotein particle remodeling [GO:0034375]; negative regulation of cytokine production involved in immune response [GO:0002719]; negative regulation of very-low-density lipoprotein particle remodeling [GO:0010903]
			MALDI TOF/TOF MS/MS	
Apolipoprotein B-100 (Apo B-100)	Anitua et al. 2015 (LS-MS/MS), Hermida-Hermida-Nogueira et al. 2020, Di Summa et al. 2020	3	LC–MS/MS	Low-density lipoprotein particle remodeling [GO:0034374]
Apolipoprotein E (Apo-E)	Anitua et al. 2015 (LS-MS/MS & TOF/TOF MS/MS), Yaprak et al. 2018, Hermida-Nogueira et al. 2020, Di Summa et al. 2020	4	LC–MS/MS,	High-density lipoprotein particle remodeling [GO:0034375]; low-density lipoprotein particle remodeling [GO:0034374]; negative regulation of blood coagulation [GO:0030195]; negative regulation of blood vessel endothelial cell migration [GO:0043537]; negative regulation of endothelial cell migration [GO:0010596]; negative regulation of endothelial cell proliferation [GO:0001937]; negative regulation of smooth muscle cell proliferation [GO:0048662]; regulation of innate immune response [GO:0045088]; very-low-density lipoprotein particle remodeling [GO:0034372]
			MALDI TOF/TOF MS/MS	
Apolipoprotein M (Apo-M; Protein G3a)	Anitua et al. 2015 (LS-MS/MS), Hermida-Nogueira et al. 2020, Di Summa et al. 2020	3	LC–MS/MS	High-density lipoprotein particle remodeling [GO:0034375]
Beta-2-glycoprotein 1 (APC inhibitor; B2GPI)	Anitua et al. 2015 (TOF/TOF MS/MS), Yaprak et al. 2018, Hermida-Nogueira et al. 2020, Di Summa et al. 2020	4	LC–MS/MS,	Blood coagulation, intrinsic pathway [GO:0007597]; blood coagulation, intrinsic pathway [GO:0007597]; chylomicron remodeling [GO:0034371]; negative regulation of angiogenesis [GO:0016525]; negative regulation of blood coagulation [GO:0030195]; negative regulation of endothelial cell migration [GO:0010596]; negative regulation of endothelial cell proliferation [GO:0001937]; positive regulation of blood coagulation [GO:0030194]; very-low-density lipoprotein particle remodeling [GO:0034372]
			MALDI TOF/TOF MS/MS	
Beta-actin-like protein 2 (Kappa-actin)	Anitua et al. 2015 (LS-MS/MS), Hermida-Nogueira et al. 2020, Di Summa et al. 2020	3	LC–MS/MS	
BH3-interacting domain death agonist (p22 BID)	Anitua et al. 2015 (LS-MS/MS), Hermida-Nogueira et al. 2020, Di Summa et al. 2020	3	LC–MS/MS	Regulation of epithelial cell proliferation [GO:0050678]; regulation of T cell proliferation [GO:0042129]
Carboxypeptidase N subunit 2	Anitua et al. 2015 (LS-MS/MS), Hermida-Nogueira et al. 2020, Di Summa et al. 2020	3	LC–MS/MS	
CD5 antigen-like (Apoptosis inhibitor expressed by macrophages; CT-2; IgM-associated peptide; SP-alpha)	Yaprak et al. 2018, Hermida-Nogueira et al. 2020, Di Summa et al. 2020	3	LC–MS/MS,	Immune system process [GO:0002376]
			MALDI TOF/TOF MS/MS	
Ceruloplasmin (Cuproxidase ceruloplasmin; Ferroxidase ceruloplasmin; Glutathione peroxidase ceruloplasmin)	Anitua et al. 2015 (LS-MS/MS & TOF/TOF MS/MS), Yaprak et al. 2018, Hermida-Nogueira et al. 2020, Di Summa et al. 2020	4	LC–MS/MS,	
			MALDI TOF/TOF MS/MS	
Clusterin (Aging-associated gene 4 protein) (Apolipoprotein J; Complement cytolysis inhibitor; Testosterone-repressed prostate message 2	Anitua et al. 2015 (LS-MS/MS), Yaprak et al. 2018, Hermida-Nogueira et al. 2020	3	LC–MS/MS,	Immune complex clearance [GO:0002434]; innate immune response [GO:0045087]; microglial cell proliferation [GO:0061518]; regulation of cell population proliferation [GO:0042127]
			MALDI TOF/TOF MS/MS	
Complement C1q subcomponent subunit C	Anitua et al. 2015 (LS-MS/MS), Hermida-Nogueira et al. 2020, Di Summa et al. 2020	3	LC–MS/MS	Complement activation, classical pathway [GO:0006958]; immune response [GO:0006955]; innate immune response [GO:0045087]
Complement C3 (C3 and PZP-like alpha-2-macroglobulin domain-containing protein 1)	Anitua et al. 2015 (LS-MS/MS), Anitua et al. 2015 (TOF/TOF MS/MS), Yaprak et al. 2018, Hermida-Nogueira et al. 2020, Di Summa et al. 2020	4	LC–MS/MS,	Immune response [GO:0006955]; neuron remodeling [GO:0016322]; positive regulation of angiogenesis [GO:0045766]
			MALDI TOF/TOF MS/MS	
Complement C4-A (Acidic complement C4; C3 and PZP-like alpha-2-macroglobulin domain-containing protein 2)	Anitua et al. 2015 (LS-MS/MS), Yaprak et al. 2018, Hermida-Nogueira et al. 2020	3	LC–MS/MS,	Complement activation [GO:0006956]; complement component C1q complex binding [GO:0001849]; innate immune response [GO:0045087]
			MALDI TOF/TOF MS/MS	
Complement factor H (H factor 1)	Anitua et al. 2015 (LS-MS/MS), Hermida-Nogueira et al. 2020, Di Summa et al. 2020	3	LC–MS/MS	Complement activation [GO:0006956]; complement component C3b binding [GO:0001851]
Fibrinogen alpha chain	Anitua et al. 2015 (LS-MS/MS), Hermida-Nogueira et al. 2020, Di Summa et al. 2020	3	LC–MS/MS	Adaptive immune response [GO:0002250]; extracellular matrix structural constituent [GO:0005201]; adaptive immune response [GO:0002250]; blood coagulation, common pathway [GO:0072377]; blood coagulation, fibrin clot formation [GO:0072378]; innate immune response [GO:0045087]
Galectin-related protein (Galectin-like protein; Lectin galactoside-binding-like protein)	Anitua et al. 2015 (LS-MS/MS), Hermida-Nogueira et al. 2020, Di Summa et al. 2020	3	LC–MS/MS	
Gelsolin (AGE; Actin-depolymerizing factor)	Anitua et al. 2015 (LS-MS/MS), Hermida-Nogueira et al. 2020, Di Summa et al. 2020	3	LC–MS/MS	
Haptoglobin (Zonulin)	Anitua et al. 2015 (LS-MS/MS & TOF/TOF MS/MS), Yaprak et al. 2018, Hermida-Nogueira et al. 2020	3	LC–MS/MS,	Acute-phase response [GO:0006953]; immune system process [GO:0002376]
			MALDI TOF/TOF MS/MS	
Heat shock protein beta-1 (HspB1; Stress-responsive protein 27)	Anitua et al. 2015 (LS-MS/MS), Hermida-Nogueira et al. 2020, Di Summa et al. 2020	3	LC–MS/MS	Positive regulation of angiogenesis [GO:0045766]; positive regulation of blood vessel endothelial cell migration [GO:0043536]
Hemoglobin subunit alpha (Alpha-globin)	Anitua et al. 2015 (LS-MS/MS), Hermida-Nogueira et al. 2020, Di Summa et al. 2020	3	LC–MS/MS	
Hemoglobin subunit beta (Beta-globin)	Anitua et al. 2015 (LS-MS/MS), Hermida-Nogueira et al. 2020, Di Summa et al. 2020	3	LC–MS/MS	
Hemoglobin subunit delta (Delta-globin)	Anitua et al. 2015 (LS-MS/MS), Hermida-Nogueira et al. 2020, Di Summa et al. 2020	3	LC–MS/MS	
Hemopexin (Beta-1B-glycoprotein)	Anitua et al. 2015 (LS-MS/MS & TOF/TOF MS/MS), Yaprak et al. 2018, Hermida-Nogueira et al. 2020, Di Summa et al. 2020	4	LC–MS/MS,	Positive regulation of humoral immune response mediated by circulating immunoglobulin [GO:0002925]
			MALDI TOF/TOF MS/MS	
Histidine-rich glycoprotein (Histidine-proline-rich glycoprotein) (HPRG)	Anitua et al. 2015 (LS-MS/MS), Hermida-Nogueira et al. 2020, Di Summa et al. 2020	3	LC–MS/MS	Angiogenesis [GO:0001525]; angiogenesis [GO:0001525]; antimicrobial humoral immune response mediated by antimicrobial peptide [GO:0061844]; negative regulation of angiogenesis [GO:0016525]; negative regulation of blood vessel endothelial cell migration [GO:0043537]; negative regulation of cell population proliferation [GO:0008285]; positive regulation of blood vessel remodeling [GO:2000504]; positive regulation of immune response to tumor cell [GO:0002839]; regulation of blood coagulation [GO:0030193]
Histone H4	Anitua et al. 2015 (LS-MS/MS), Hermida-Nogueira et al. 2020, Di Summa et al. 2020	3	LC–MS/MS	
Immunoglobulin heavy constant alpha 1 (Ig alpha-1 chain C region)	Anitua et al. 2015 (LS-MS/MS), Hermida-Nogueira et al. 2020, Di Summa et al. 2020	3	LC–MS/MS	Adaptive immune response [GO:0002250]; adaptive immune response [GO:0002250]; immune response [GO:0006955]
Immunoglobulin heavy constant gamma 1 (Ig gamma-1 chain C region)	Anitua et al. 2015 (LS-MS/MS & TOF/TOF MS/MS), Yaprak et al. 2018, Hermida-Nogueira et al. 2020	3	LC–MS/MS,	Adaptive immune response [GO:0002250]
			MALDI TOF/TOF MS/MS	
Immunoglobulin heavy constant gamma 2 (Ig gamma-2 chain C region)	Anitua et al. 2015 (LS-MS/MS), Yaprak et al. 2018, Hermida-Nogueira et al. 2020	3	LC–MS/MS,	Adaptive immune response [GO:0002250]
			MALDI TOF/TOF MS/MS	
Immunoglobulin heavy constant mu (Ig mu chain C region)	Anitua et al. 2015 (LS-MS/MS), Yaprak et al. 2018, Hermida-Nogueira et al. 2020, Di Summa et al. 2020	4	LC–MS/MS,	Adaptive immune response [GO:0002250]; adaptive immune response [GO:0002250]; innate immune response [GO:0045087]
			MALDI TOF/TOF MS/MS	
Immunoglobulin kappa constant (Ig kappa chain C region)	Anitua et al. 2015 (LS-MS/MS), Yaprak et al. 2018, Hermida-Nogueira et al. 2020, Di Summa et al. 2020	4	LC–MS/MS	Adaptive immune response [GO:0002250]; adaptive immune response [GO:0002250]; immune response [GO:0006955]; immunoglobulin mediated immune response [GO:0016064]
			MALDI TOF/TOF MS/MS	
Inter-alpha-trypsin inhibitor heavy chain H1 (ITI heavy chain H1; Serum-derived hyaluronan-associated protein)	Anitua et al. 2015 (LS-MS/MS), Hermida-Nogueira et al. 2020, Di Summa et al. 2020	3	LC–MS/MS	
Inter-alpha-trypsin inhibitor heavy chain H4 (ITI heavy chain H4; Plasma kallikrein sensitive glycoprotein 120)	Anitua et al. 2015 (LS-MS/MS), Yaprak et al. 2018, Hermida-Nogueira et al. 2020, Di Summa et al. 2020	4	LC–MS/MS,	Acute-phase response [GO:0006953]
			MALDI TOF/TOF MS/MS	
Keratin, type I cytoskeletal 10 (Cytokeratin-10)	Anitua et al. 2015 (LS-MS/MS), Hermida-Nogueira et al. 2020, Di Summa et al. 2020	3	LC–MS/MS	
Keratin, type I cytoskeletal 9 (Cytokeratin-9)	Anitua et al. 2015 (LS-MS/MS), Hermida-Nogueira et al. 2020, Di Summa et al. 2020	3	LC–MS/MS	
Keratin, type II cytoskeletal 1 (CK-1; Hair alpha protein)	Anitua et al. 2015 (LS-MS/MS), Hermida-Nogueira et al. 2020, Di Summa et al. 2020	3	LC–MS/MS	Complement activation, lectin pathway [GO:0001867]; regulation of angiogenesis [GO:0045765]
Kininogen-1 (Alpha-2-thiol proteinase inhibitor; Fitzgerald factor)	Anitua et al. 2015 (LS-MS/MS & TOF/TOF MS/MS), Hermida-Nogueira et al. 2020, Di Summa et al. 2020	3	LC–MS/MS,	Blood coagulation [GO:0007596]; blood coagulation [GO:0007596]; negative regulation of blood coagulation [GO:0030195]
			MALDI TOF/TOF MS/MS	
PDZ and LIM domain protein 1 (Elfin)	Anitua et al. 2015 (LS-MS/MS), Hermida-Nogueira et al. 2020, Di Summa et al. 2020	3	LC–MS/MS	
Peptidyl-prolyl cis-trans isomerase A (PPIase A; Cyclophilin A)	Anitua et al. 2015 (LS-MS/MS), Hermida-Nogueira et al. 2020, Di Summa et al. 2020	3	LC–MS/MS	
Peroxiredoxin-1 (Natural killer cell-enhancing factor A; Proliferation-associated gene protein; Thioredoxin peroxidase 2)	Anitua et al. 2015 (LS-MS/MS), Hermida-Nogueira et al. 2020, Di Summa et al. 2020	3	LC–MS/MS	Cell population proliferation [GO:0008283]; fibroblast proliferation [GO:0048144]
Phosphatidylinositol-glycan-specific phospholipase D (PI-G PLD)	Anitua et al. 2015 (LS-MS/MS), Hermida-Nogueira et al. 2020, Di Summa et al. 2020	3	LC–MS/MS	Cell migration involved in sprouting angiogenesis [GO:0002042]; cell migration involved in sprouting angiogenesis [GO:0002042]; hematopoietic stem cell migration [GO:0035701]; hematopoietic stem cell migration to bone marrow [GO:0097241]; negative regulation of cell population proliferation [GO:0008285]; positive regulation of endothelial cell migration [GO:0010595]
Plasma protease C1 inhibitor (C1 Inh; Serpin G1)	Anitua et al. 2015 (LS-MS/MS), Yaprak et al. 2018, Hermida-Nogueira et al. 2020	3	LC–MS/MS,	Blood coagulation [GO:0007596]; innate immune response [GO:0045087]
			MALDI TOF/TOF MS/MS	
Profilin-1 (Epididymis tissue protein Li 184a)	Anitua et al. 2015 (LS-MS/MS), Hermida-Nogueira et al. 2020, Di Summa et al. 2020	3	LC–MS/MS	Positive regulation of epithelial cell migration [GO:0010634]
Protein AMBP (Protein HC)	Anitua et al. 2015 (LS-MS/MS & TOF/TOF MS/MS), Yaprak et al. 2018, Hermida-Nogueira et al. 2020, Di Summa et al. 2020	4	LC–MS/MS,	Negative regulation of immune response [GO:0050777]
			MALDI TOF/TOF MS/MS	
Protein S100-A9 (Calgranulin-B; Calprotectin L1H subunit; Leukocyte L1 complex heavy chain; Migration inhibitory factor-related protein 14)	Anitua et al. 2015 (LS-MS/MS), Hermida-Nogueira et al. 2020, Di Summa et al. 2020	3	LC–MS/MS	Antimicrobial humoral immune response mediated by antimicrobial peptide [GO:0061844]; antimicrobial humoral immune response mediated by antimicrobial peptide [GO:0061844]; endothelial cell migration [GO:0043542]; innate immune response [GO:0045087]
Prothrombin (Coagulation factor II)	Anitua et al. 2015 (LS-MS/MS), Yaprak et al. 2018, Hermida-Nogueira et al. 2020, Di Summa et al. 2020	4	LC–MS/MS,	Acute-phase response [GO:0006953]; antimicrobial humoral immune response mediated by antimicrobial peptide [GO:0061844]; blood coagulation [GO:0007596]; negative regulation of blood coagulation [GO:0030195]; positive regulation of blood coagulation [GO:0030194]; positive regulation of cell population proliferation [GO:0008284]; regulation of blood coagulation [GO:0030193]; response to wounding [GO:0009611]
			MALDI TOF/TOF MS/MS	
Ras-related protein Rap-1b (GTP-binding protein smg p21B)	Anitua et al. 2015 (LS-MS/MS), Hermida-Nogueira et al. 2020, Di Summa et al. 2020	3	LC–MS/MS	Cell population proliferation [GO:0008283]
Serotransferrin (Transferrin; Beta-1 metal-binding globulin)	Anitua et al. 2015 (LS-MS/MS & TOF/TOF MS/MS), Yaprak et al. 2018, Hermida-Nogueira et al. 2020, Di Summa et al. 2020	4	LC–MS/MS,	
			MALDI TOF/TOF MS/MS	
Serum amyloid P-component (SAP; 9.5S alpha-1-glycoprotein)	Anitua et al. 2015 (LS-MS/MS & TOF/TOF MS/MS), Hermida-Nogueira et al. 2020, Di Summa et al. 2020	3	LC–MS/MS,	Acute-phase response [GO:0006953]; innate immune response [GO:0045087]; negative regulation of wound healing [GO:0061045]
			MALDI TOF/TOF MS/MS	
Talin-1	Anitua et al. 2015 (LS-MS/MS), Hermida-Nogueira et al. 2020, Di Summa et al. 2020	3	LC–MS/MS	
Thrombospondin-1 (Glycoprotein G)	Anitua et al. 2015 (LS-MS/MS), Hermida-Nogueira et al. 2020, Di Summa et al. 2020	3	LC–MS/MS	Cell migration [GO:0016477]; immune response [GO:0006955]; negative regulation of angiogenesis [GO:0016525]; negative regulation of blood vessel endothelial cell migration [GO:0043537]; negative regulation of blood vessel endothelial cell proliferation involved in sprouting angiogenesis [GO:1903588]; negative regulation of cell migration involved in sprouting angiogenesis [GO:0090051]; negative regulation of cell population proliferation [GO:0008285]; negative regulation of endothelial cell migration [GO:0010596]; negative regulation of endothelial cell proliferation [GO:0001937]; negative regulation of sprouting angiogenesis [GO:1903671]; positive regulation of angiogenesis [GO:0045766]; positive regulation of blood coagulation [GO:0030194]; positive regulation of blood vessel endothelial cell migration [GO:0043536]; positive regulation of cell migration [GO:0030335]; positive regulation of cell population proliferation [GO:0008284]; positive regulation of endothelial cell migration [GO:0010595]; positive regulation of smooth muscle cell proliferation [GO:0048661]; sprouting angiogenesis [GO:0002040]
Transthyretin (ATTR; Prealbumin; TBPA)	Anitua et al. 2015 (LS-MS/MS & TOF/TOF MS/MS), Yaprak et al. 2018, Hermida-Nogueira et al. 2020, Di Summa et al. 2020	4	LC–MS/MS,	
			MALDI TOF/TOF MS/MS	
Trem-like transcript 1 protein (TLT-1; Triggering receptor expressed on myeloid cells-like protein 1)	Anitua et al. 2015 (LS-MS/MS), Hermida-Nogueira et al. 2020, Di Summa et al. 2020	3	LC–MS/MS	Innate immune response [GO:0045087]
Tropomyosin alpha-4 chain (TM30p1; Tropomyosin-4)	Yaprak et al. 2018, Hermida-Nogueira et al. 2020, Di Summa et al. 2020	3	LC–MS/MS,	
			MALDI TOF/TOF MS/MS	
Vitamin D-binding protein (VDB; Gc protein-derived macrophage activating factor)	Anitua et al. 2015 (LS-MS/MS & TOF/TOF MS/MS), Yaprak et al. 2018, Hermida-Nogueira et al. 2020	3	LC–MS/MS,	
			MALDI TOF/TOF MS/MS	
Vitronectin (VN; Serum-spreading factor)	Anitua et al. 2015 (LS-MS/MS), Yaprak et al. 2018, Hermida-Nogueira et al. 2020, Di Summa et al. 2020	4	LC–MS/MS,	Cell migration [GO:0016477]; extracellular matrix organization [GO:0030198]; immune response [GO:0006955]; liver regeneration [GO:0097421]; negative regulation of blood coagulation [GO:0030195]; positive regulation of smooth muscle cell migration [GO:0014911]; positive regulation of wound healing [GO:0090303]
			MALDI TOF/TOF MS/MS	

An overview of the above MS/MS studies shows that there are 72 common proteins that are present in at least three studies, and among them, 20 proteins are present in all four studies (Table 1). Based on the gene ontology (GO; geneontology.org) characteristics, these proteins are widely involved with various stages of wound healing, including hemostasis/coagulation (prothrombin, fibrinogen, antithrombin, kininogen, alpha-2-antiplasmin, and vitronectin), inflammation (alpha-1-antitrypsin, alpha-1-antichymotrypsin, haptoglobin, serum amyloid P, Immunoglobulins, complement factors, clusterin, annexin A1, and S100-A9), proliferation and angiogenesis [actin, profilin-1, adenylyl cyclase-associated protein 1 (CAP1), heat shock protein beta-1, apolipoprotein A-I, angiotensinogen, and Rap-1b], remodeling and ECM organization (vitronectin, thrombospondin-1, actin cytoskeleton proteins, gelsolin, inter-alpha-trypsin inhibitor, and apolipoproteins), and serve as antioxidants (Table 1). These proteins are also known to regulate various fibroblasts functions. For example, thrombospondin-1 is reported to modulate fibroblast proliferation and ECM organization (62, 63). Angiotensinogen is a precursor that is enzymatically converted into angiotensin II, which not only plays a role in endothelial cell migration, but also regulates fibroblast proliferation and collagen deposition (64, 65). Peroxiredoxin-1 acts as an antioxidant enzyme that protects fibroblasts against oxidative stress, supporting their survival and proliferation (66). Vitronectin, as a component of both the blood plasma and ECM, mediates cell-matrix interactions that are important for fibroblast adhesion and migration (67, 68). S100-A9 is highly expressed in neutrophils and macrophages and plays a role in the fibroblast-macrophage interaction that drives inflammatory signaling, leading to fibroblast activation and differentiation into myofibroblasts (69, 70).

The limitations in the dynamic range of the mass spectrometry approaches do not always allow to identify low abundance proteins (e.g., certain enzymes, hormones, chemokines, cytokines and growth factors) along with the major other proteins, including certain matrix components, in the same samples. Thus, using only mass spectrometric approaches, certain very abundant proteins maybe over-represented while some biologically significant low abundance molecules may remain undetected. Therefore, use of immunoassays to specifically look for these low abundance molecules are important to complement the proteomics profiling. It is likely that when proteomics techniques are further developed, more accurate picture of the entire proteome of PRF preparations may emerge.

## Regulation of fibroblast functions by PRF

Most molecular studies about PRF have focused on mapping the growth factors and cytokines present in the biomaterial rather than the fibroblast response to them. In addition, nothing is known about effects of PRF on specific fibroblast subpopulations. As discussed above, the composition of PRF is very complex, making it difficult to decipher functions of its individual components on fibroblasts. Therefore, it is useful to study the total effect of all bioactive components in the PRF preparations that are present or released at the same time. This is because various bioactive factors may have synergistic, additive, inhibitory or opposite effects when presented to the cells at the same time. Furthermore, the target fibroblasts need to express appropriate cell surface receptors that recognize and bind these molecules. The repertoire of surface receptor expression differs between different fibroblast populations (20, 71), which may result to selective activation of certain fibroblasts over others. In any case, the clinical success of PRF is thought to depend on the storm of concentrated bioactive molecules derived from PRF that boost and supplement effect of these and other molecules already present in the acute wounds. In addition, as the cells and their secretions are trapped in the fibrin-rich PRF matrix the release of bioactive factors is sustained over several days (9, 14, 51, 56). This is considered to contribute to the regenerative potential of PRF by potently stimulating responsive recipient cells, including fibroblasts, over a prolonged period, although the exact mechanisms are not well understood.

*In vitro studies*—While some effects of PRF on fibroblasts *in vivo* can be indirect and mediated by PRF-activated other cells, including immune cells, *in vitro* studies are useful to study direct interactions between PRF and fibroblasts. In order to understand whether the blood concentrate alone can elicit similar response as normal wound healing in gingival fibroblasts, we compared gene expression in L-PRF-treated cultured human gingival fibroblasts over time to gene expression in human and porcine gingival wounds at the early wound healing phases (72). The changes in target gene expression in the early wounds were very similar to those observed in the fibroblasts treated with L-PRF, including a decrease in *COL1A1* (A1 chain of collagen 1) expression and significant increases in *IL1B* (interleukin 1 beta), *IL6* (interleukin 6), *IL8* (interleukin 8), *MMP1* (matrix metalloproteinase 1), *MMP3* (matrix metalloproteinase 3), *FN1* (fibronectin) and *TNC* (tenascin C) compared to unwounded tissue. Remarkably, the expression of cytokines in L-PRF-treated fibroblasts was 200–800-fold higher compared to non-treated control cells, which indicates that PRF strongly enhances fibroblast immunomodulatory functions and paracrine cell-to-cell communication with other key cells in wound healing (72). For example, IL-1β plays an anti-bacterial role in oral wound healing by providing protection against bacterial insults (73). IL-6 supports wound healing by facilitating fibroblast proliferation, keratinocyte migration, macrophage recruitment, and new blood vessel formation (74). Additionally, IL-8 may aid wound healing by boosting keratinocyte proliferation and migration while decreasing wound contraction (75). It is well established that both epithelial and mesenchymal cell migration is modulated by fibronectin and TN-C (76). MMPs degrade the structural components of the ECM to break down matrix barriers to also facilitate cell migration and regulate inflammation by activating or deactivating inflammatory mediators (77, 78). Upregulation of MMPs occurs in the early stages of scarless wound healing in adult gingiva and fetal skin suggesting that their early activity may determine the wound healing outcome (79, 80). Therefore, it is interesting that in L-PRF-treated fibroblasts, MMP-1 and -3 were significantly up-regulated at the gene and protein levels by PRF at an early time point but down-regulated at a later time point (10, 72).

TGF-β, which has been consistently found in PRF, is one of the key growth factors involved in wound healing, and plays an important role in immunomodulation and promoting various functions of fibroblasts, including ECM deposition (81). Recent studies have provided evidence that PRF activates TGF-β signaling in target cells. Di Summa et al. (61) demonstrated activation of the canonical Smad-dependent pathway in human gingival fibroblasts by PRF, showing phosphorylation and nuclear translocation of Smad2/3 and TGF-β-mediated induction of target genes, such as *IL11* (interleukin 11), *NOX4* (NADPH oxidase 4), and *PRG4* (proteoglycan 4, lubricin) (61). Kargarpour et al. (82) directly compared PRF with unfractionated blood clots and also found that both preparations activated TGF-β/Smad signaling in gingival fibroblasts, while also exerting comparable anti-inflammatory effects (82). Bi et al. (72) examined the broader transcriptional and signaling responses of gingival fibroblasts to L-PRF. They also confirmed Smad3 phosphorylation, but also identified activation of non-canonical pathways, including ERK1/2, JNK, and p38 MAPKs, which were functionally necessary for many of the observed gene expression changes (72). These findings suggest that TGF-β released from PRF remains biologically active and signals via the canonical Smad and non-canonical MAPK pathways to regulate fibroblast functions. In addition to soluble mediators, fibroblast functions could also be regulated by direct cell-cell contact with various immune cells contained within PRF during wound healing. These interactions could also modulate inflammation and homing of immune cells to the wound site (83). However, nothing is known whether such cell-cell interactions are important in wound healing of PRF-treated tissue sites.

Matrix components of PRF may also modulate cell functions important for wound healing. For instance, fibrin, the major structural component of PRF, supports cell adhesion, binds, stores and releases bioactive molecules and promotes angiogenesis (14, 84–86). Cell culture experiments have shown that PRF also induces proliferation of human microvascular endothelial cells and vascular-like tube formation of human umbilical vein endothelial cells indicating pro-angiogenetic function (72, 87). Interestingly, molecules released from human gingival fibroblasts that were pre-treated with L-PRF also promoted endothelial cell tube formation compared to releasate from non-L-PRF treated fibroblasts (72). Accordingly, L-PRF induced expression of angiogenesis-related genes *VEGFA* and *FGF2* in fibroblasts (10, 72). Therefore, PRF may not only promote angiogenesis by containing pro-angiogenic soluble factors and fibrin, but also enhances the function of fibroblasts to promote angiogenesis. Since blood supply is crucial for wound healing, the pro-angiogenic functions of PRF are expected to aid in the early healing process.

Cell culture studies exploring effects of PRF on fibroblasts have considerable heterogeneity due to use of different PRF preparation methods and fibroblasts. Although, comparison studies are still limited, some studies using distinct PRF preparations [PRP, L-PRF, Alb-PRF, and i-PRF (a liquid formulation of injectable PRF)] or fibroblasts from different tissues show a general ability of these concentrates to promote key fibroblast functions, including proliferation (88). In addition, standard scratch wound closure or chemotaxis assays have been used to study effect of PRF preparations on fibroblast migration, an important process in wound healing. While these assays do not recapitulate all aspects of fibroblast migration in a complex three-dimensional matrix *in vivo*, they show that PRF releasate can promote movement and chemotaxis by fibroblasts (16, 72, 87, 89–91). PRP also promotes skin fibroblast collagen synthesis and expression of *MMPs* (90), while Alb-PRF has similar effect to other PRF preparations to stimulate gingival fibroblast expression of *TGFB1* and *COL1A1,* albeit with an extended effect up to 7 days (16). Imani et al. (92) compared early (6 h) gene expression changes in gingival fibroblasts treated with PRF membrane lysates and PRF serum (liquid exudate released during membrane preparation) to non-treated cells by bulk RNA sequencing. Compared to controls, PRF membranes and serum induced significant gene expression changes in 404 and 94 genes, respectively, indicating a more pronounced effect induced by the PRF membranes. Among the commonly upregulated 46 genes were many inflammatory mediators [*CXCL1, CXCL5, CXCL6, CXCL8, IL33, IL6, PTGS2/COX2* (prostaglandin-endoperoxide synthase 2)], and *STC1* (stanniocalcin-1; involved in calcium metabolism). There were only 14 commonly downregulated genes, including *IFIT1*, *IFIT2*, and *IFIT3* (interferon-induced protein with tetratricopeptide repeats; involved in innate immune response) and *OSR1* and *OSR2* (odd skipped-related transcription factors; involved in Wnt-signaling and development), *FGF18* (fibroblast growth factor 18; involved in development and tissue repair) and *GDF15* (growth differentiation factor 15; involved in TGF-β signaling). Thus, depending on protocols used, different PRF preparations may have partially different effects on fibroblasts. However, in general they appear to promote fibroblast recruitment, induce fibroblasts to produce an immunomodulatory microenvironment and support their ECM deposition and remodeling functions.

Apart from different PRF preparation methods, differences in PRF quality may result in distinct fibroblast responses (10). A 6-minute delay in processing of PRF by centrifugation leads to reduced expression of *FGF2*, *MMP1*, *MMP3*, *IL6*, and *IL8* in gingival fibroblasts treated with releasates from this PRF compared to PRF processed without delay (10). Interestingly, this effect is gene-specific because up-regulation of *VEGFA* and *FN1-EDA* (cellular fibronectin), and down-regulation of *CTSK* (cathepsin K), *CXCL12*, and *TGFB1* showed no different between the two treatment groups (10, 72). Delay in centrifugation causes depletion of leukocytes and platelets (10) suggesting that optimal viability of these cells is required for some of the specific effects of PRF.

*In vivo studies*—There is a lack of studies that have directly addressed regulation of fibroblast functions by PRF *in vivo*. Therefore, assessing effects of PRF on these cells needs to be deduced based on known roles of fibroblasts in wound healing. For instance, a prospective cohort study about the use of L-PRF in treatment of various refractory ulcers, including venous leg ulcers, diabetic foot ulcers, pressure ulcers, and complex wounds, showed significant improvements in wound closure after the L-PRF therapy (93). Fibroblast function is impaired in poorly healing wounds, which may at least in part explain delayed wound healing observed in patients. A diabetic mouse model showed that, compared to dermal fibroblasts from control mice, there was a 75% reduction in fibroblast migration with an increase in MMP9 and decrease in VEGF production in the diabetic animals (94). Similarly, high glucose levels impair proliferation and migration of human gingival fibroblasts (95). Since PRF is well documented in promoting fibroblast migration and angiogenesis, it may counteract the impaired function of fibroblasts in these poorly healing wounds. However, more studies are needed to assess cell type-specific effects of PRF on fibroblasts in wound healing *in vivo*.

## Limitations and current gaps

Despite growing evidence that PRF enhances fibroblast-mediated wound healing, several key gaps in knowledge remain. First, this far studies have focused on standard heterogenous fibroblast cultures, overlooking the well-established fibroblast heterogeneity between tissues and existence of fibroblast subpopulations with distinct immunomodulatory, angiogenic, or ECM remodeling potency. It is unknown whether PRF selectively activates regenerative fibroblast phenotypes and which components of PRF maybe important in this process. Second, the precise mechanisms by which PRF regulates fibroblast behavior are not fully defined, as the relative contributions of its soluble bioactive molecules, EVs, fibrin scaffold, and leukocyte–fibroblast interactions remain unclear. Third, direct *in vivo* evidence of fibroblast-specific responses to PRF is limited, with most conclusions inferred from overall clinical outcomes rather than mechanistic cellular analyses; advanced approaches such as lineage tracing or single-cell RNA sequencing have not been applied to PRF-treated wounds. Additionally, wide variability in PRF preparation protocols leads to inconsistent bioactive composition and cellular effects, and there is no consensus on the optimal formulation, dosage, or application timing to target fibroblasts. Finally, the temporal and tissue-specific dynamics of PRF-fibroblast interactions remain poorly understood, including how long PRF influences fibroblasts *in vivo* and whether its effects differ between oral mucosa, skin, or chronic wounds. Addressing these gaps is essential to optimizing PRF formulations, improving reproducibility, and fully harnessing its regenerative potential.

## Conclusions and future directions

**Figure 1:**
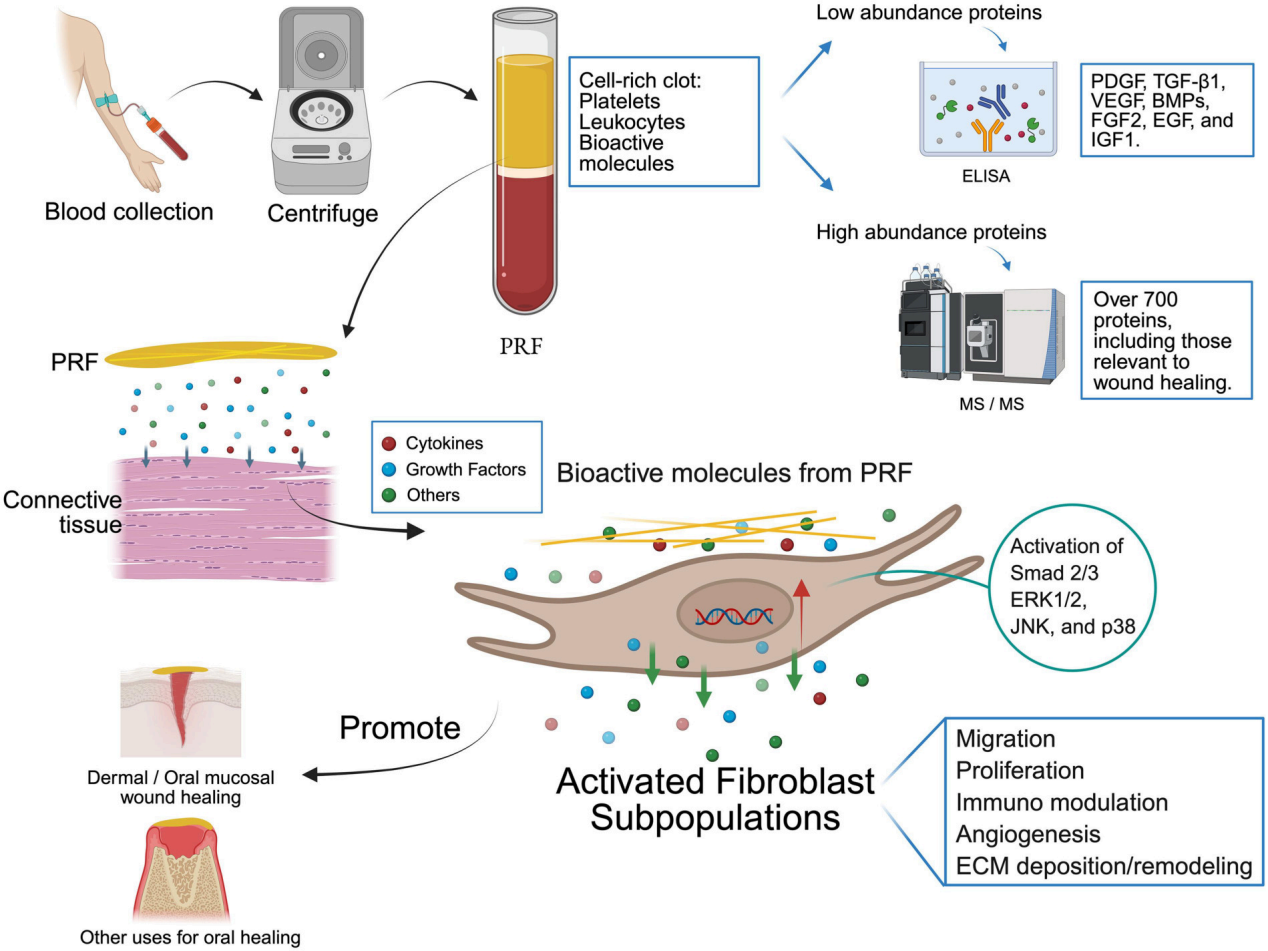
The graphical abstract of the effects of platelet-rich fibrin (PRF) on fibroblasts. Created in BioRender. Bi, J. (2026) https://BioRender.com/wjchht8

PRF is an autologous blood-derived biomaterial that includes components that modulate fibroblast functions critical for wound healing. Fibroblasts, including various subpopulations, play a significant role in the wound healing process. Cell culture studies have shown that PRF enhances various fibroblast functions, including proliferation and migration, and modulates expression of various genes and proteins important in immune modulation, angiogenesis, ECM deposition and remodeling (Figure 1). It also activates both the canonical TGF-β/Smad and non-canonical MAPK (ERK, p38, JNK) pathways in fibroblasts. Proteomic studies reveal that PRF contains numerous proteins involved in coagulation, immune regulation, angiogenesis, and ECM organization, many of which can directly affect fibroblast behavior. However, the preparation protocols of PRF currently in use for various clinical applications have varied greatly. There is evidence that these different protocols, while having some common effects on fibroblasts, alter PRF's cellular and molecular composition, biological effects and functional potency.

A recent publication by Anitua et al. (2024) showed that PRGF promotes the regeneration ability of the periodontal ligament stem cells (96). Gingival fibroblast cultures and gingival MSCs exhibit the capacity for trilineage differentiation. Future research could focus on dissecting the fibroblast-specific effects of PRF using advanced research tools such as single-cell RNA sequencing, as well as lineage tracing and spatial transcriptomics, to determine whether PRF preferentially activates pro-regenerative fibroblast subpopulations. Also, mechanistic studies separating the contributions of soluble factors, extracellular vesicles, and the fibrin matrix will be essential to identify the key components responsible for its bioactivity. Clinically, combining PRF with biomaterials or drugs that target specific fibroblast subpopulations may offer a promising strategy to enhance its regenerative potential. However, well-designed controlled clinical trials are necessary to identify the ideal PRF types, dosing strategies, and timing for different wound types across oral, cutaneous, and chronic injury settings.
